# Supplementary material for: Aspergillus fumigatus calcium-responsive transcription factors regulate cell wall architecture promoting stress tolerance, virulence and caspofungin resistance
Source: PLoS Genet. 2019 Dec 30;15(12):e1008551. doi: 10.1371/journal.pgen.1008551 (PMC6948819; doi:10.1371/journal.pgen.1008551)
Supplement: S1 Fig — BlastP analyses using the mature ZipD protein sequence were performed, at the expected (e-value) cut-off thresholds of 1.0e-5, 1.0e-50 and 1.0e-100, on the predicted proteomes of 791 fungi, representing the 13 different taxonomic classes or subphyla within Dikarya, as presented on the JGI Mycocosm portal. Diagrammatic representation of the fungal tree of life (not indicative of evolutionary time) adapted from the JGI MycoCosm. Pie charts show the number of species genomes within each taxonomic class with at least one protein similar to ZipD at the indicated BlastP thresholds. (PDF) [file pgen.1008551.s001.pdf]

A.

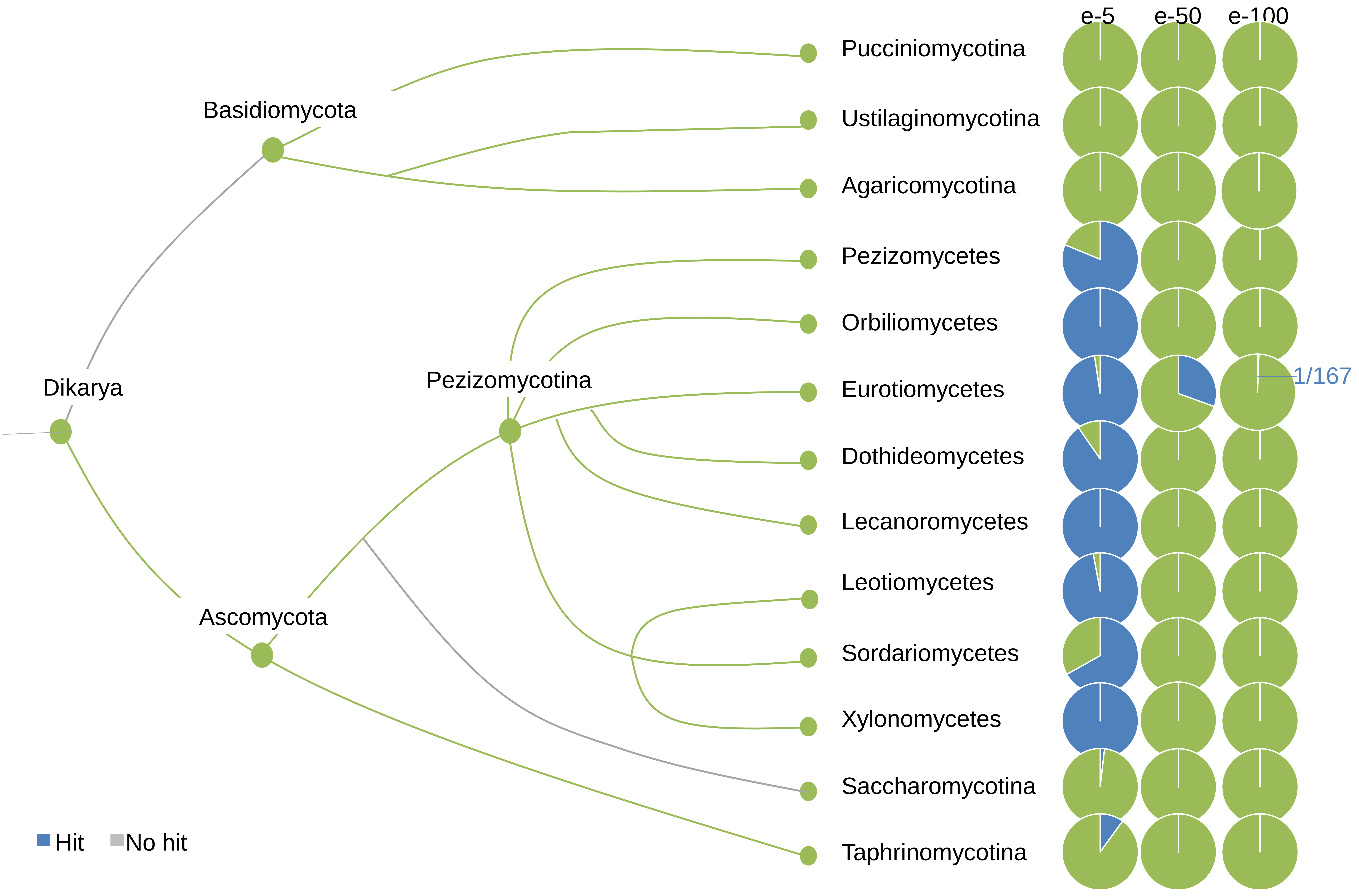
e-5
e-50
e-100
Basidiomycota
Dikarya
Pezizomycotina
Ascomycota
Pucciniomycotina
Ustilaginomycotina
Agaricomycotina
Pezizomycetes
Orbiliomycetes
Eurotiomycetes
Dothideomycetes
Lecanoromycetes
Leotiomycetes
Sordariomycetes
Xylonomycetes
Saccharomycotina
Taphrinomycotina
1/167
Hit
No hit

B.

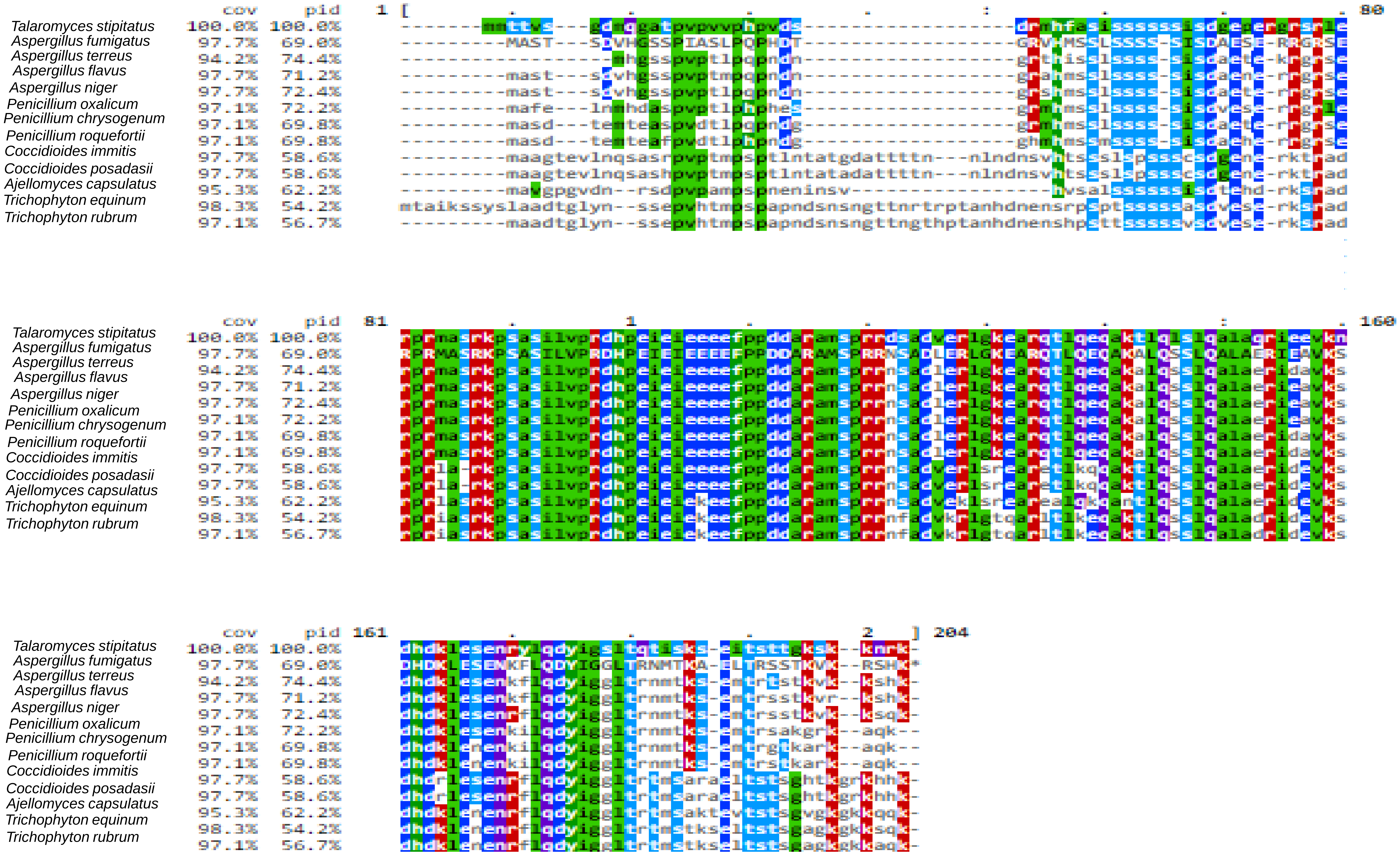
cov
pid
Talaromyces stipitatus
Aspergillus fumigatus
Aspergillus terreus
Aspergillus flavus
Aspergillus niger
Penicillium oxalicum
Penicillium chrysogenum
Penicillium roquefortii
Coccidioides immitis
Coccidioides posadasii
Ajellomyces capsulatus
Trichophyton equinum
Trichophyton rubrum
1
80
81
160
161
204

Supplementary Figure S1 - The distribution ZipD related proteins throughout the fungal tree of life. (A) BlastP analyses using the mature ZipD protein sequence were performed, at the expected (e-value) cut-off thresholds of 1.0e-5, 1.0e-50 and 1.0e-100, on the predicted proteomes of 791 fungi, representing the 13 different taxonomic classes or subphyla within Dikarya, as presented on the JGI Mycocosm portal. Diagrammatic representation of the fungal tree of life (not indicative of evolutionary time) adapted from the JGI MycoCosm. Pie charts show the number of species genomes within each taxonomic class with at least one protein similar to ZipD at the indicated BlastP thresholds. (B) Multiple sequence alignment by using Clustal Omega (https://www.ebi.ac.uk/Tools/msa/clustalo/) and Mview (https://www.ebi.ac.uk/Tools/msa/mview/) of selected species of Eurotiomycetes. Sequences and Blastp alignments were obtained from https://www.yeastgenome.org/blastfungal?uploadFile=&program=blastp#XP_002150417.1.
When compared with *A. fumigatus*: *A. oryzae* [e-value=2e-67, identity=92 %, score=207 bits (527)], *A. flavus* [e-value=2e-67, identity=92%, score=207 bits (527)], *A. niger* [e-value=2e-64, identity=92 %, score=199 bits (507)], *A. terreus* [e-value=2e-61, identity=90%, score=191 bits (486)], *P. chrysogenum* [e-value=7e-55, identity=84%, score=175 bits (444)], *P. oxalicum* [e-value=1e-54, identity=87%, score=174 bits (442)], *P. roquefortii* [e-value=2e-49, identity=82%, score= 161 bits (407)], *T. stipitatus* [e-value=1e-39, identity=78%, score=207 bits (344), *C. immitis* [e-value=1e-38, identity= 80%, score=134 bits (338), *C. posadasii* (e-value=2e-38, identity= 80%, score=138 (338), *T. equinum* [e-value=2e-36, identity= 78 %, score=129 bits (325), *T. rubrum* [e-value=5e-36, identity=82%, score=128 bits (321), and *A. capsulatus* [e-value=5e-36, identity=80%, score= 127 bits (320).
