# Supplementary figures and images for: Aspergillus fumigatus calcium-responsive transcription factors regulate cell wall architecture promoting stress tolerance, virulence and caspofungin resistance
Source: PLoS Genet. 2019 Dec 30;15(12):e1008551. doi: 10.1371/journal.pgen.1008551 (PMC6948819; doi:10.1371/journal.pgen.1008551)

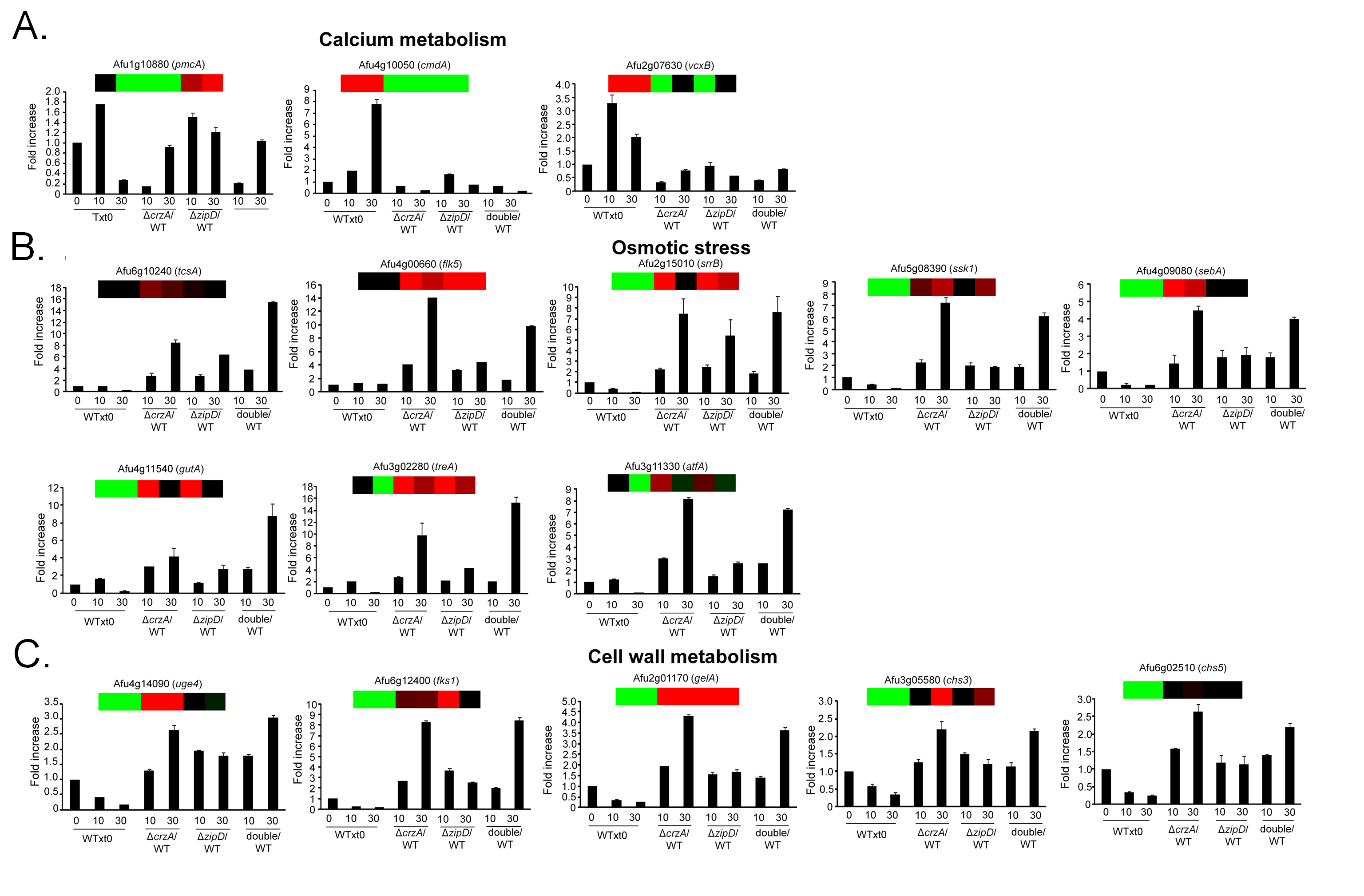

Supplement: S2 Fig — Gene expression was normalized using cofA (Afu5g10570). Standard deviations present the average of three independent biological repetitions (each with 2 technical repetitions). Above each graph the corresponding result of the heat-map RNAseq for each gene. Once more, the wild-type is shown as 10 and 30 min calcium stress versus time zero (20 hours growth), and gene deletion strains are shown as the deletion strain versus the equivalent wild-type 10 and 30 min time points (the mutant values have been normalised to the basal level of expression of each gene before stress, i.e., expression ratios are being compared: wild-type 10 min versus time zero divided by a specific mutant 10 and 30 min versus time zero). The expression of these sixteen genes showed a high level of correlation with the RNA-seq data (Pearson correlation from 0.7055 to 0.9187; Fig 4E). (TIF) [file pgen.1008551.s002.tif]

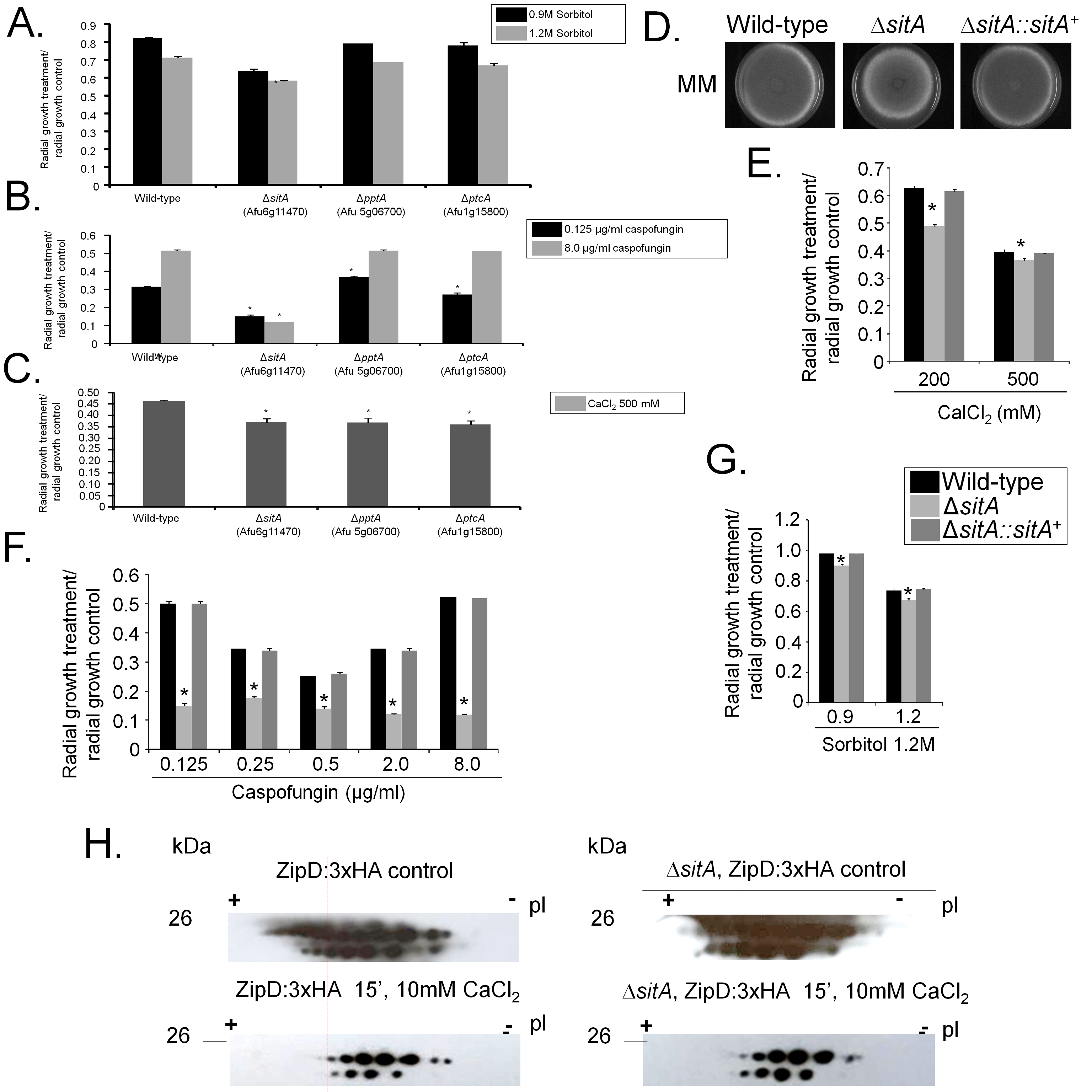

Supplement: S5 Fig — Screening for the phosphatase mutants more sensitive to sorbitol (A), caspofungin (B), and CaCl2 (C). (TIF) [file pgen.1008551.s005.tif]

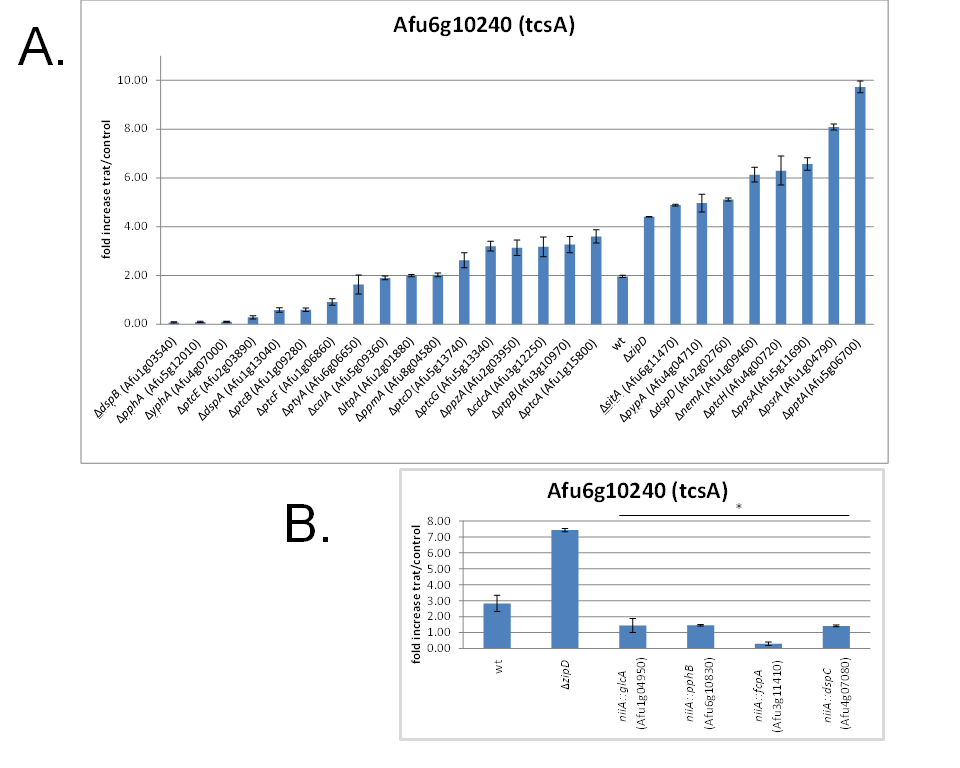

Supplement: S6 Fig — (A) The wild-type, ΔzipD, and all phosphatase catalytic subunit null mutants were grown for 16 h at 37°C and transferred to 200 mM CaCl2 for 0 and 10 mins. Gene expression was normalized using cofA (Afu5g10570). Standard deviations present the average of three independent biological repetitions (each with 2 technical repetitions). Statistical analysis was performed using a one-way ANOVA test when compared to the wild-type condition (*p<0.05). (B) The wild-type, ΔzipD, and four conditional were grown for 16 h at 37°C in MM+nitrate as a single nitrogen source, and then transferred to MM+ammonium tartrate as a single nitrogen source, and subsequently to 200 mM CaCl2 for 0 and 10 mins. Gene expression was normalized using cofA (Afu5g10570). Standard deviations present the average of three independent biological repetitions (each with 2 technical repetitions). Statistical analysis was performed using a one-way ANOVA test when compared to the wild-type condition (*p<0.05). (TIF) [file pgen.1008551.s006.tif]
