# Supplementary material for: Aspergillus fumigatus calcium-responsive transcription factors regulate cell wall architecture promoting stress tolerance, virulence and caspofungin resistance
Source: PLoS Genet. 2019 Dec 30;15(12):e1008551. doi: 10.1371/journal.pgen.1008551 (PMC6948819; doi:10.1371/journal.pgen.1008551)
Supplement: S3 Fig — (B) Phenotype analysis of wild type and ZipD:3xHA strains which were grown in MM plates for 5 days at 37°C. (PDF) [file pgen.1008551.s003.pdf]

A.

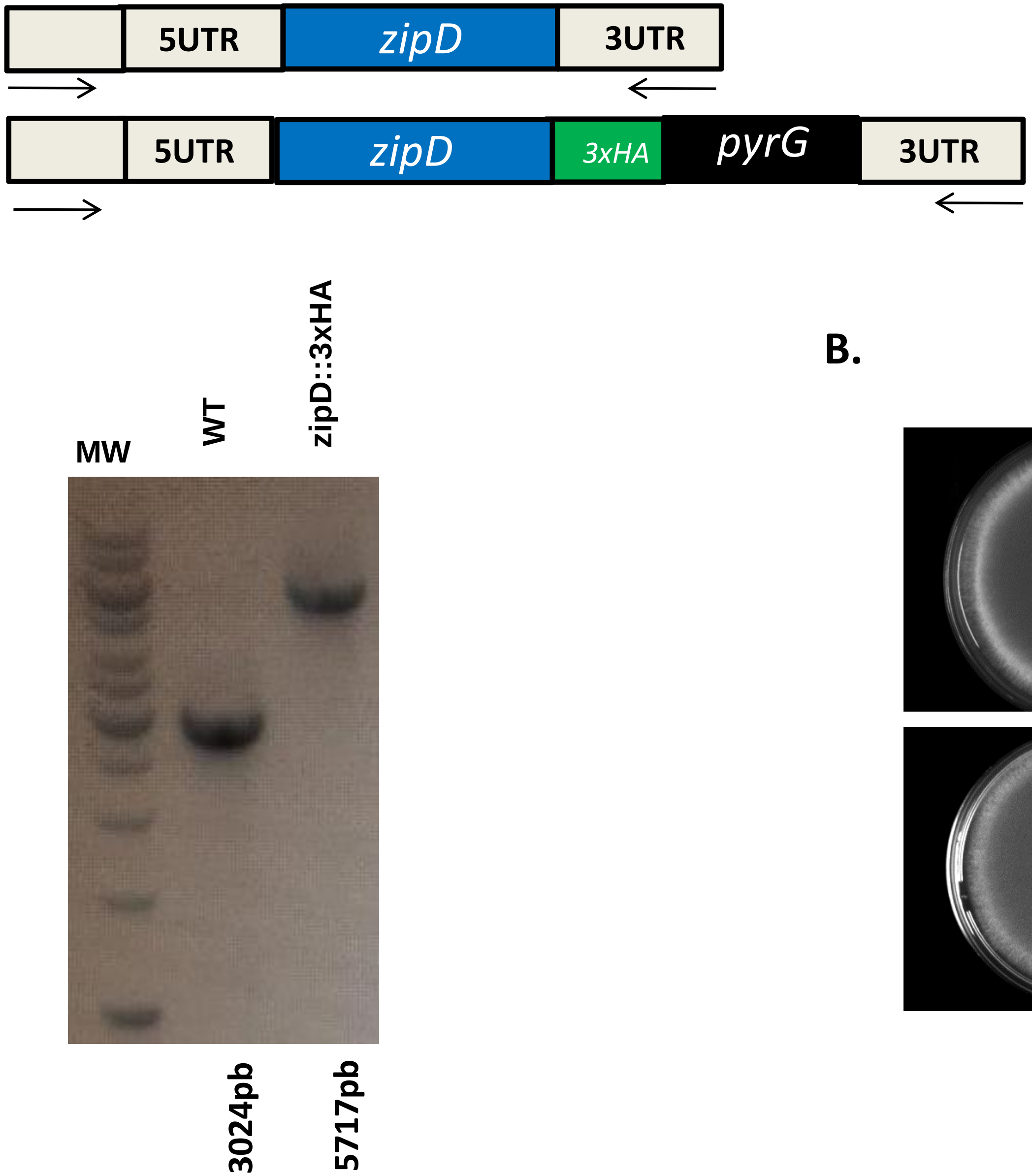

Primer forward: zipD 5fw ext
Primer reverse: zipD pRS426 3rv

B.

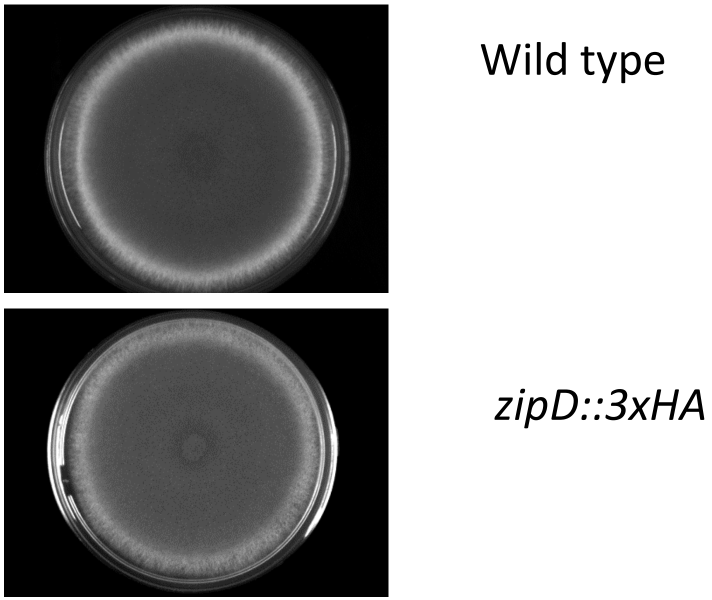

Supplementary Figure S3- PCR scheme to check the ZipD::3xHA strain. (B) Phenotype analysis of wild type and ZipD::3xHA strains which were grown in MM plates for 5 days at 37°C
