# Supplementary material for: Aspergillus fumigatus calcium-responsive transcription factors regulate cell wall architecture promoting stress tolerance, virulence and caspofungin resistance
Source: PLoS Genet. 2019 Dec 30;15(12):e1008551. doi: 10.1371/journal.pgen.1008551 (PMC6948819; doi:10.1371/journal.pgen.1008551)
Supplement: S4 Fig — (A) PCR scheme to verify the homologous integration of CalA::GFP and CalA::GFP ZipD:3xHA. (B) Phenotypic analysis of wild type, CalA::GFP (candidate 2 in the PCR) and CalA::GFP ZipD:3xHA (candidate 2 in the PCR) strains which were grown in YAG plates, with or without CaCl2 for 3 days at 37°C. (C) Verification of interaction between CalA and ZipD by Co-IP. Affinity purification assays from GFP‐tagged CalA strain in the background of 3xHA‐tagged ZipD were performed with (a) GFP‐Trap and (b) anti‐HA beads to verify interactions. The coimmunoprecipitated proteins were analysed by the indicated antibodies. (PDF) [file pgen.1008551.s004.pdf]

A.

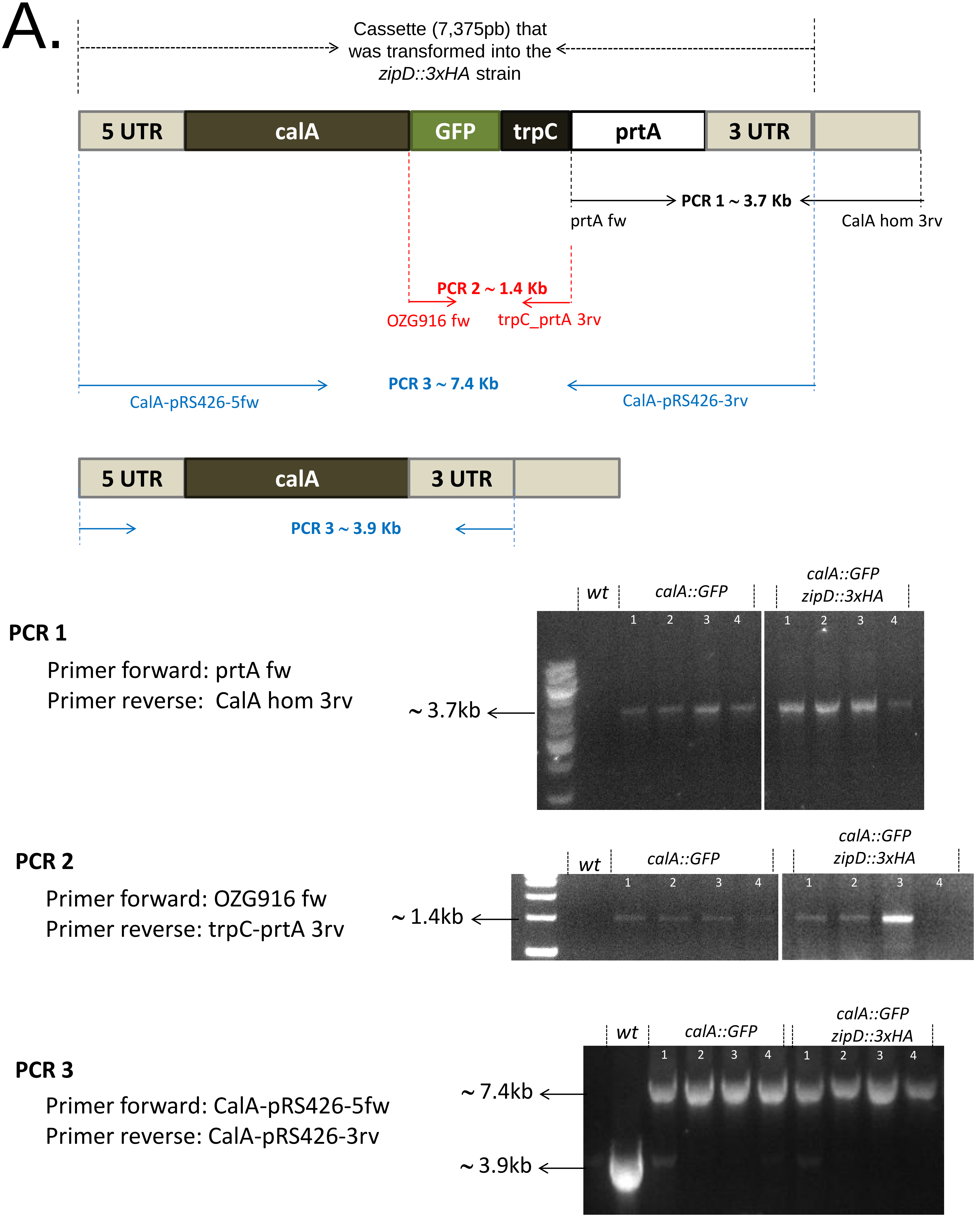

Cassette (7,375pb) that was transformed into the *zipD::3xHA* strain
5 UTR
calA
GFP
trpC
prtA
3 UTR
PCR 1 ~ 3.7 Kb
prtA fw
CalA hom 3rv
PCR 2 ~ 1.4 Kb
OZG916 fw
trpC_prtA 3rv
PCR 3 ~ 7.4 Kb
CalA-pRS426-5fw
CalA-pRS426-3rv
5 UTR
calA
3 UTR
PCR 3 ~ 3.9 Kb
PCR 1
Primer forward: prtA fw
Primer reverse: CalA hom 3rv
~ 3.7kb
wt
calA::GFP
calA::GFP zipD::3xHA
1 2 3 4
1 2 3 4
PCR 2
Primer forward: OZG916 fw
Primer reverse: trpC-prtA 3rv
~ 1.4kb
wt
calA::GFP
calA::GFP zipD::3xHA
1 2 3 4
1 2 3 4
PCR 3
Primer forward: CalA-pRS426-5fw
Primer reverse: CalA-pRS426-3rv
~ 7.4kb
~ 3.9kb
wt
calA::GFP
calA::GFP zipD::3xHA
1 2 3 4
1 2 3 4

B.

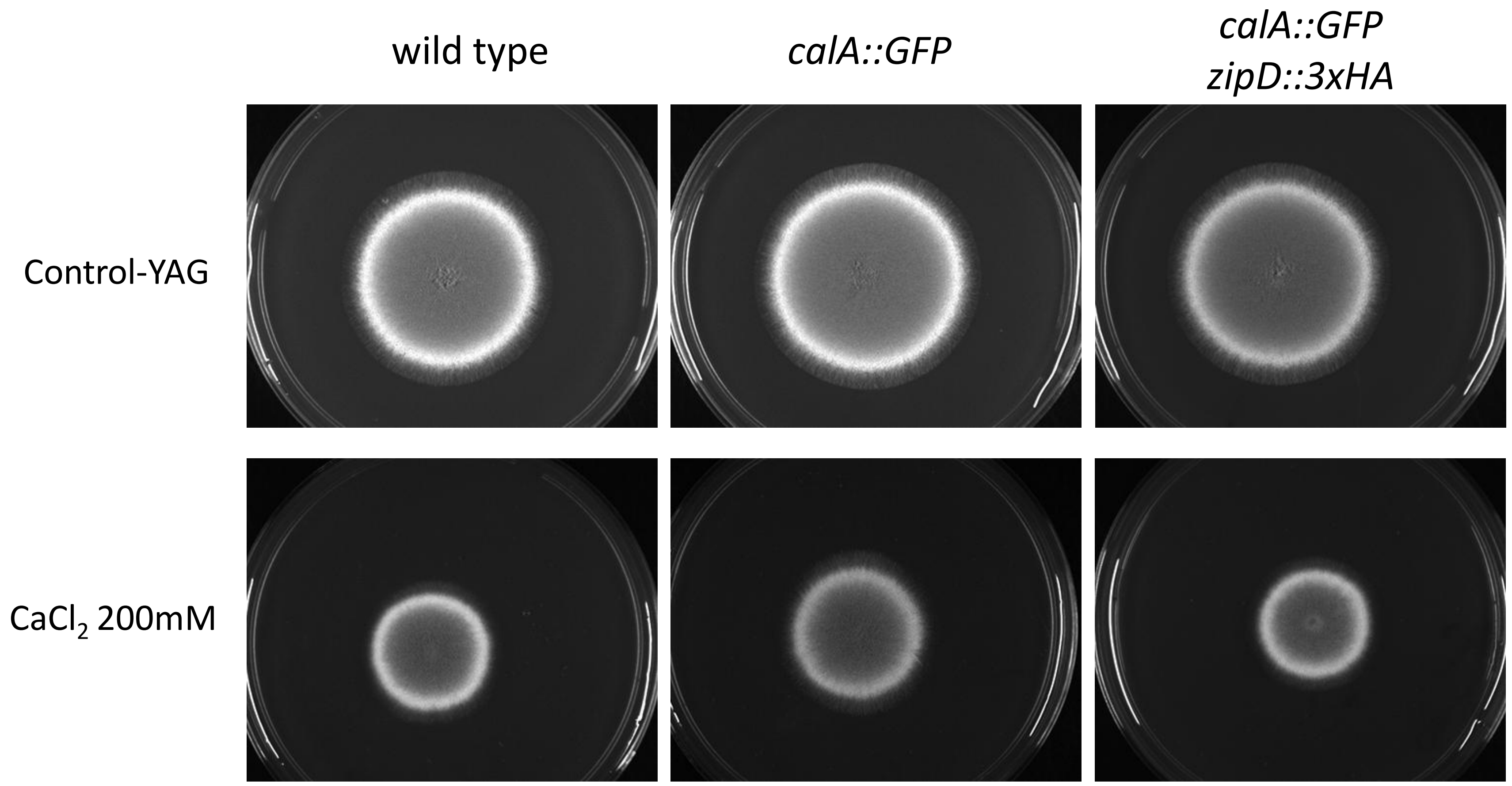
wild type
calA::GFP
calA::GFP
zipD::3xHA
Control-YAG
CaCl2 200mM

C.

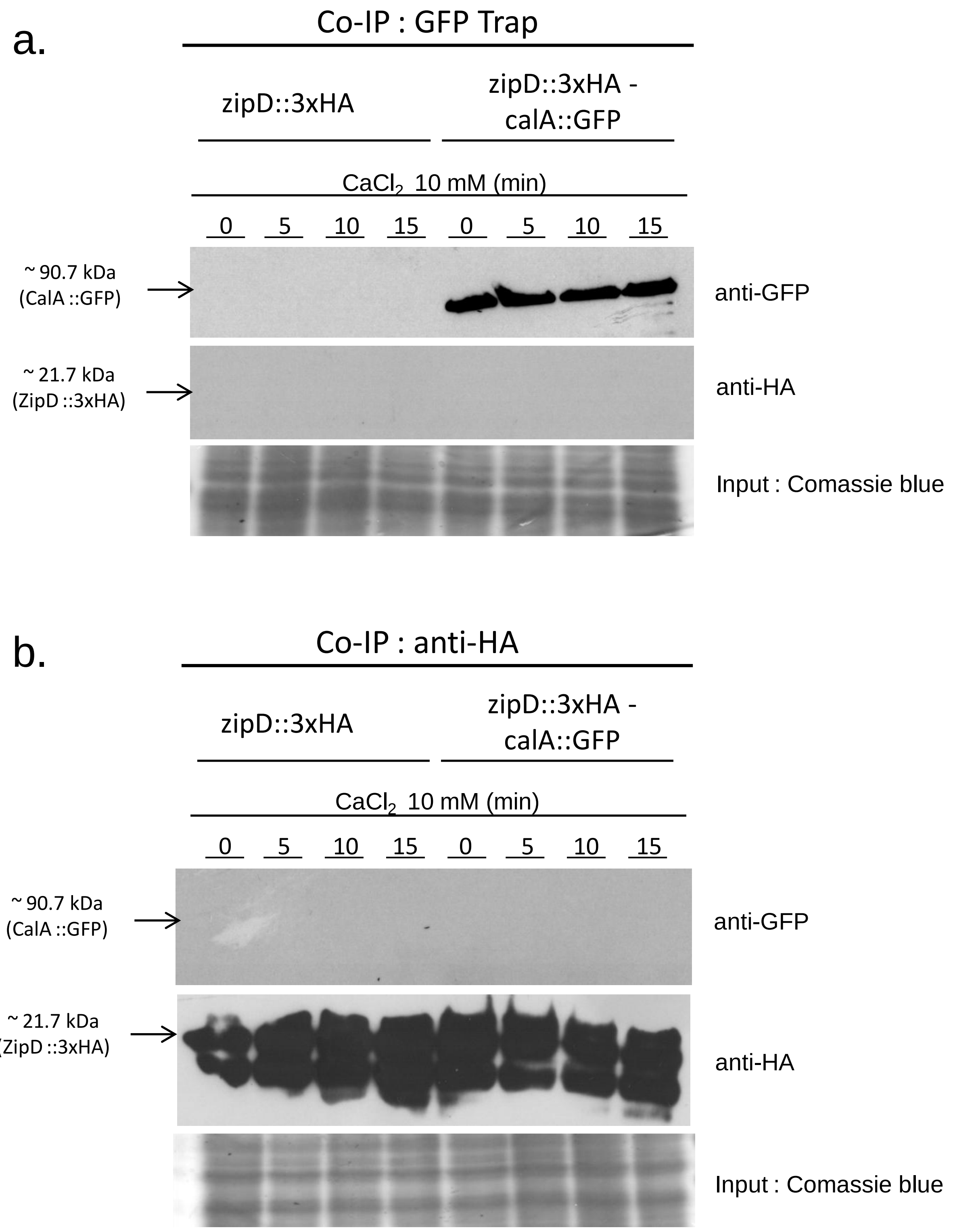

Supplementary Figure S4 – Co-Immunoprecipitation of CalA::GFP and ZipD::3xHA. (A) PCR scheme to verify the homologous integration of CalA::GFP and CalA::GFP ZipD::3xHA. (B) Phenotypic analysis of wild type, CalA::GFP (candidate 2 in the PCR) and CalA::GFP ZipD::3xHA (candidate 2 in the PCR) strains which were grown in YAG plates, with or without $CaCl_2$ for 3 days at 37°C. (C) Verification of interaction between CalA and ZipD by Co-IP. Affinity purification assays from GFP-tagged CalA strain in the background of 3xHA-tagged ZipD were performed with (a) GFP-Trap and (b) anti-HA beads to verify interactions. The coimmunoprecipitated proteins were analysed by the indicated antibodies.
