# Supplementary material for: Aspergillus fumigatus calcium-responsive transcription factors regulate cell wall architecture promoting stress tolerance, virulence and caspofungin resistance
Source: PLoS Genet. 2019 Dec 30;15(12):e1008551. doi: 10.1371/journal.pgen.1008551 (PMC6948819; doi:10.1371/journal.pgen.1008551)
Supplement: S10 Table — (DOCX) [file pgen.1008551.s016.docx]

Supplementary Table S10 – *Aspergillus fumigatus* phosphatase mutants.

| Family^a^ | Subfamily^b^ | *A. fumigatus*  ID genes | *A. fumigatus*  Proteins | Effect of the deletion  on *A. fumigatus* | *S. cerevisiae* protein |
| --- | --- | --- | --- | --- | --- |
| S/T | PPP | [Afu5g12010](http://www.aspergillusgenome.org/cgi-bin/locus.pl?locus=Afu5g12010) | Δ*pphA* | Viable | Pph3p |
| S/T | PPP | [Afu1g04950](http://www.aspergillusgenome.org/cgi-bin/locus.pl?locus=Afu1g04950) | *niiA::glcA* | Lethal | Glc7p |
| S/T | PPP | [Afu6g11470](http://www.aspergillusgenome.org/cgi-bin/locus.pl?locus=Afu6g11470) | Δ*sitA* | Viable | Sit4P |
| S/T | PPP | [Afu2g03950](http://www.aspergillusgenome.org/cgi-bin/locus.pl?locus=Afu2g03950) | Δ*ppzA* | Viable | Ppz1p |
| S/T | PPP | [Afu6g10830](http://www.aspergillusgenome.org/cgi-bin/locus.pl?locus=Afu6g10830) | *niiA::pphB* | Lethal | Pph21p |
| S/T | PPP | [Afu5g06700](http://www.aspergillusgenome.org/cgi-bin/locus.pl?locus=Afu5g06700) | Δ*pptA* | Viable | Ppt1p |
| S/T | PPP | [Afu5g09360](http://www.aspergillusgenome.org/cgi-bin/locus.pl?locus=Afu5g09360) | Δ*calA*/Δ*cnaA* | Viable | Cmp2p |
| S/T | PPM | [Afu1g15800](http://www.aspergillusgenome.org/cgi-bin/locus.pl?locus=Afu1g15800) | Δ*ptcA* | Viable | Ptc6p |
| S/T | PPM | [Afu1g09280](http://www.aspergillusgenome.org/cgi-bin/locus.pl?locus=Afu1g09280) | Δ*ptcB* | Viable | Ptc2p |
| S/T | PPM | [Afu8g04580](http://www.aspergillusgenome.org/cgi-bin/locus.pl?locus=Afu8g04580) | Δ*ppmA* | Viable | -/- |
| S/T | PPM | Afu5g13740,  Afu2g03890 | Δ*ptcD*,  Δ*ptcE* | Both viable | Ptc2p |
| S/T | PPM | [Afu1g06860](http://www.aspergillusgenome.org/cgi-bin/locus.pl?locus=Afu1g06860) | Δ*ptcF* | Viable | Ptc5p |
| S/T | PPM | [Afu5g13340](http://www.aspergillusgenome.org/cgi-bin/locus.pl?locus=Afu5g13340) | Δ*ptcG* | Viable | Ptc1p |
| S/T | PPM | Afu4g00720 | Δ*ptcH* | Viable | Ptc1p |
| S/T | Asp-based | [Afu1g09460](http://www.aspergillusgenome.org/cgi-bin/locus.pl?locus=Afu1g09460) | Δ*nemA* | Viable | Nem1p |
| S/T | Asp-based | [Afu3g11410](http://www.aspergillusgenome.org/cgi-bin/locus.pl?locus=Afu3g11410) | *niiA:fcpA* | Lethal | Fcp1p |
| S/T | Asp-based | [Afu1g04790](http://www.aspergillusgenome.org/cgi-bin/locus.pl?locus=Afu1g04790) | Δ*psrA* | Viable | Psr1p |
| PTP | Dual-specificity | [Afu5g11690](http://www.aspergillusgenome.org/cgi-bin/locus.pl?locus=Afu5g11690) | Δ*ppsA* | Viable | -/- |
| PTP | Dual-specificity | Afu4g07080 | Δ*dspC* | Lethal | -/- |
| PTP | Dual-specificity | [Afu2g02760](http://www.aspergillusgenome.org/cgi-bin/locus.pl?locus=Afu2g02760) | Δ*dspD* | Viable | -/- |
| PTP | Dual-specificity | [Afu3g12250](http://www.aspergillusgenome.org/cgi-bin/locus.pl?locus=Afu3g12250) | Δ*cdcA* | Viable | Cdc14p |
| PTP | Dual-specificity | [Afu1g13040](http://www.aspergillusgenome.org/cgi-bin/locus.pl?locus=Afu1g13040) | *niiA::dspA* | Viable | -/- |
| PTP | Dual-specificity | Afu1g03540 | Δ*dspB* | Viable | -/- |
| PTP | Classical | [Afu3g10970](http://www.aspergillusgenome.org/cgi-bin/locus.pl?locus=Afu3g10970) | Δ*ptpB* | Viable | Ptp1p |
| PTP | Classical | [Afu4g04710](http://www.aspergillusgenome.org/cgi-bin/locus.pl?locus=Afu4g04710) | Δ*pypA* | Viable | Ptp2p |
| PTP | LMW-PTP | [Afu2g01880](http://www.aspergillusgenome.org/cgi-bin/locus.pl?locus=Afu2g01880) | Δ*ltpA* | Viable | Ltp1p/Yvh1p |
| PTP | - | [Afu4g07000](http://www.aspergillusgenome.org/cgi-bin/locus.pl?locus=Afu4g07000) | Δ*yphA* | Viable | -/- |
| PTP | - | Afu6g06650 | Δ*ptyA* | Viable | Ptc7p |

^a^Family abbreviations: S/T, serine/threonine; PTP, protein tyrosine phosphatase.

^b^Subfamily abbreviations: PPP, phosphoprotein phosphatase; PPM, Mg2+ or Mn2+-dependent protein phosphatase; Asp-based,

aspartate-based phosphatase; LMW-PTP, low-molecular-weight protein tyrosine phosphatase
